# Duration of mechanical ventilation in trauma patients: risk factor for VAP?

**DOI:** 10.1186/cc14390

**Published:** 2015-03-16

**Authors:** I Turriziani, A Cecchi, A Giugni, L Copertino

**Affiliations:** 1 Ospedale Maggiore, Bologna, Italy

## Introduction

In the literature, duration of mechanical ventilation (DMV) is often considered an important risk factor (RF) [1] for VAP [2] in critical patients; generally the whole duration of MV is taken into account, including days before and after infection onset. We tried to assess whether, counting only MV days prior to VAP development (MVp), something would change.

## Methods

We considered, in a 10-year period, data prospectively collected in our database (4D solution, V11) on trauma patients admitted to the ICU directly from the emergency department. Inclusion criteria were: age ≥16 years, ICU length of stay (ICUlos) ≥4 days, DMV ≥48 hours; we excluded patients who received antibiotics before VAP (or during the whole stay, for patients without VAP) and with incomplete data. Data were: age, sex, prehospital GCS <9, prehospital intubation (preHTI), admission base excess (BE), Injury Severity Score (ISS), surgery, massive transfusion, feeding, antacids, nursing, DMV, ICUlos and MVp. MVp was calculated as the difference between the first day of VAP and the first day of MV in patients who developed VAP (vapY) and whole DMV in patients that did not (vapN). We only considered the first infectious episode. The outcome was VAP onset. Group comparison was made with Fisher's exact test and Student's *t *test. Significant variables were evaluated in a logistic regression (LR) model; the Hosmer-Lemeshow test (HL) was used as the post-estimation test. Odds ratio (OR) and 95% confidence interval (95% CI) were calculated. Statistical significance for *P *< 0.05. We used Stata/IC 10.1 for analysis.

## Results

A total of 541 patients met the inclusion criteria, 378 (69.9%) developed VAP. MVp does not seem to be a RF for VAP because they are longer in vapN than in vapY (mean MVp 5.5 vs. 4.41, *P *= 0.001). PreHTI (vapY/N: 49.74%/38.65%; OR: 1.57; 95% CI: 1.08 to 2.28), ISS (mean vapY/N: 28.4/25.55; *P *= 0.0018), BE (mean vapY/N: -3.76/-3.04; *P *= 0.03) were significantly different between the two groups. In LR only preHTI (OR: 1.47; 95% CI: 1.01 to 2.15) and ISS (OR: 1.03; 95% CI: 1.01 to 1.05) are RF for VAP (HL: *P *= 0.133).

## Conclusion

In our study MVp are not a RF for VAP in trauma patients, although the whole DMV is longer in patients with VAP (mean DMV vapY/N: 13.57/6.09; *P *= 0.0001). Further studies could confirm whether the whole DMV in trauma patients with VAP is a consequence of infection.
